# The High-Resolution Structure Reveals Remarkable Similarity in PD-1 Binding of Cemiplimab and Dostarlimab, the FDA-Approved Antibodies for Cancer Immunotherapy

**DOI:** 10.3390/biomedicines10123154

**Published:** 2022-12-06

**Authors:** Tae-Jun Jeong, Hyun-Tae Lee, Nahyeon Gu, Yu-Jeong Jang, Seung-Beom Choi, Ui-Beom Park, Sang-Hyung Lee, Yong-Seok Heo

**Affiliations:** Department of Chemistry, Konkuk University, 120 Neungdong-ro, Gwangjin-gu, Seoul 05029, Republic of Korea

**Keywords:** antibody, cancer immunotherapy, cemiplimab, crystal structure, dostarlimab, immune checkpoint, nivolumab, PD-1, pembrolizumab

## Abstract

Multiple tumors have responded well to immunotherapies, which use monoclonal antibodies to block the immune checkpoint proteins and reactivate the T-cell immune response to cancer cells. Significantly, the anti-PD-1 antibodies pembrolizumab and nivolumab, which were approved in 2014, have revolutionized cancer therapy, demonstrating dramatic improvement and longer duration. The US FDA authorized the third anti-PD-1 medication, cemiplimab, in 2018 for use in patients with cutaneous squamous cell carcinoma. To further understand the molecular mechanism of the antibody drug, we now reveal the intricate structure of PD-1 in complex with the cemiplimab Fab at a resolution of 1.98 Å. The cemiplimab–PD-1 interaction preoccupies the space for PD-L1 binding with a greater binding affinity than the PD-1/PD-L1 interaction, which is the basis for the PD-1 blocking mechanism. The structure reveals that cemiplimab and dostarlimab are significantly similar in PD-1 binding, although the precise interactions differ. A comparative investigation of PD-1 interactions with the four FDA-approved antibodies reveals that the BC, C’D, and FG loops of PD-1 adopt distinct conformations for optimal interaction with the antibodies. The structural characteristics in this work could be helpful information for developing more potent anti-PD-1 biologics against cancer.

## 1. Introduction

Antibody-mediated blockade of immune checkpoint proteins has been unprecedentedly successful in treating multiple tumors [1]. Programmed cell death-1 (PD-1), a co-inhibitory receptor within the immunological synapse, is mainly expressed in immune cells such as T cells, B cells, dendritic cells, and NK cells [2]. PD-1 is a crucial immune checkpoint for maintaining immune tolerance and exhaustion of T cells in a tumor, restricting active immune responses against cancer cells [3]. The ligands of PD-1, PD-L1 and PD-L2, are highly expressed in tumor cells, enabling the tumor cells to escape from T-cell immune responses by interacting with PD-1 [4]. Inhibition of the interaction of PD-1 with its ligands using monoclonal antibodies can restore T-cell immune responses against cancer cells [5]. The US Food and Drug Administration (FDA) has approved eight monoclonal antibodies against immunological checkpoint proteins, including PD-1, PD-L1, and cytotoxic T-lymphocyte protein 4 (CTLA-4), with four approvals for anti-PD-1, three for anti-PD-L1, and one for anti-CTLA-4. Unfortunately, the overall response rate of these immune checkpoint inhibitors’ monotherapies is insufficient, even though they have significantly improved patient outcomes and prolonged patient survival for certain forms of cancer. In order to increase the therapeutic efficiency and overall response rate, various immune checkpoint molecules, such as the lymphocyte activation gene 3 protein (LAG3), T-cell immunoreceptor with Ig and ITIM domains (TIGIT), and T-cell immunoglobulin mucin receptor 3 (Tim-3), have been highlighted as attractive combination targets with the antibodies against PD-1, PD-L1, and CTLA-4 [6,7]. As a result, numerous investigational antibodies targeting these immune checkpoints are currently undergoing clinical trials and showing encouraging results in patients with a variety of malignancies [8,9]. 

Currently, four FDA-approved antibodies targeting PD-1 are clinically available for cancer immunotherapy. Nivolumab (OPDIVO^®^) and pembrolizumab (KEYTRUDA^®^) were both approved by the US FDA in 2014 to treat non-small cell lung cancer and melanoma [10,11,12]. Since then, monotherapy or drug combinations have expanded the use of the antibodies for various tumors, including renal cell carcinoma, classical Hodgkin lymphoma, head and neck squamous cell carcinoma, urothelial carcinoma, esophageal carcinoma, endometrial cancer, squamous cell carcinoma, hepatocellular carcinoma, and breast cancer [13]. In 2018, cemiplimab (LIBTAYO^®^) was approved for treating squamous cell carcinoma [14]. Cemiplimab was also approved by the FDA as the first immunotherapy for patients with advanced basal cell carcinoma and first-line treatment of advanced non-small cell lung cancer with PD-L1 of at least 50% in 2021 [14,15]. In addition, the FDA authorized dostarlimab (JEMPERLI^®^), the fourth anti-PD-1 antibody, for the treatment of mismatch repair-deficient (dMMR) endometrial cancer and dMMR solid tumors in 2021 [16,17]. 

Cemiplimab is a fully human and hinge-stabilized IgG4 (S228P) kappa monoclonal antibody that tightly binds to PD-1 and inhibits its binding to PD-L1 and PD-L2, preventing PD-1-mediated immune escape of cancer cells [18]. The K_D_ value of cemiplimab to human PD-1 is 0.6 nM, with an association rate constant of 3.2 × 10^5^ M^−1^s^−1^ and dissociation rate constant of 1.9 × 10^−4^ s^−1^, indicating fast association and slow dissociation [19]. Cemiplimab demonstrated similarly potent binding for cynomolgus monkey PD-1 but did not bind to rat and mouse PD-1. Cemiplimab did not cause Fc-mediated effector functions, such as antibody-dependent cellular cytotoxicity (ADCC) or complement-dependent cytotoxicity (CDC), as would be predicted for an IgG4 antibody [19]. 

Crystal structures of the FDA-approved antibodies in complex with the ectodomain of PD-1 have revealed the molecular details of their PD-1 blockade mechanism [20,21,22,23,24,25,26,27]. While nivolumab predominantly binds to the PD-1’s flexible N-terminal region, pembrolizumab primarily binds to the C’D loop [18,19,20,21]. The binding of dostarlimab to PD-1 involves the interaction with the flexible BC, C’D and FG loops within PD-1 [24]. Although PD-1 does not involve these flexible loop regions for its ligand binding, these antibodies efficiently hinder the ligand–receptor interaction by steric blocking through the interactions with these loops [25]. With a resolution of 3.40 Å, the crystal structure of PD-1 in complex with the single-chain variable fragment (scFv) of cemiplimab was just recently described, giving the structural basis for PD-1 blockade by cemiplimab [26]. Here, we present the crystal structure of human PD-1 in complex with the Fab fragment of cemiplimab at a resolution of 1.98 Å, revealing a more precise binding mechanism and epitope. A detailed analysis of the PD-1 binding of these FDA-approved antibodies enriches our understanding of the molecular basis of anti-PD-1 therapeutics for developing improved biologics and effective combination therapies for treating cancer.

## 2. Materials and Methods

### 2.1. Expression and Purification of Proteins

The details for the expression and purification of the PD-1 protein and cemiplimab Fab are described in the supplementary materials. In short, the DNA sequence encoding the extracellular domain of human PD-1 (aa 26–150) was subcloned into pET-21a. The protein was expressed in *E. coli* BL21 (DE3) cells as inclusion bodies and refolded by dialysis. The protein was purified by Ni-affinity and gel filtration chromatography. A modified pBAD vector was used for subcloning the DNA sequences for the heavy and light chains of the cemiplimab Fab, which are synthesized after codon optimization for *E. coli* expression. Ni-affinity and gel filtration chromatography were used to purify the cemiplimab Fab, which was expressed in the periplasm of *E. coli*.

### 2.2. Crystallization of the Complex

Cemiplimab’s Fab fragment was mixed with PD-1 protein at a molar ratio of 1: 1.2, and the mixture was incubated for 30 min on ice. The mixture was loaded using a buffer containing 20 mM Tris, pH 7.5, and 200 mM NaCl on a HiLoad 26/600 Superdex 200 pg column (Cytiva, Marlborough, MA, USA) for gel filtration chromatography. The purified PD-1/cemiplimab complex was concentrated to 10 mg mL^−1^ and crystallized by hanging-drop vapor diffusion with a well solution containing 0.1 M sodium HEPES pH 7.5 and 5% (*w*/*v*) PEG 3350 at 20 °C. Before the X-ray diffraction experiment, crystals were soaked into the crystallization reservoir solution plus 20% (*v*/*v*) glycerol and quickly frozen in liquid nitrogen.

### 2.3. Data Collection and Structure Determination

X-ray diffraction data of the crystal of PD-1/cemiplimab complex were acquired at Pohang Accelerator Laboratory (PAL) (Pohang, Korea) beamline 5C and automatically processed and scaled using XDS to a resolution of 1.98 Å [27]. The structures of PD-1 and the variable and constant regions of the Fab fragment in the complex structure of PD-1/pembrolizumab Fab (PDB code 5GGS) were used as search models for molecular replacement (MR) using Phaser software [20,28]. Model building and structure refinement were carried out using PHENIX and Coot [29,30]. Statistics for data collection and structure determination are presented in Table 1. All structural figures were produced using PyMol (Schrödinger, Inc., New York, NY, USA). The atomic coordinates and structure factors for the complex structure of PD-1/cemiplimab were deposited in a Protein Data Bank (http://www.rcsb.org (deposited: 21 September 2022) under the accession code 8GY5.

## 3. Results

### 3.1. Crystal Structure of PD-1 in Complex with Cemiplimab

**Figure 1:**
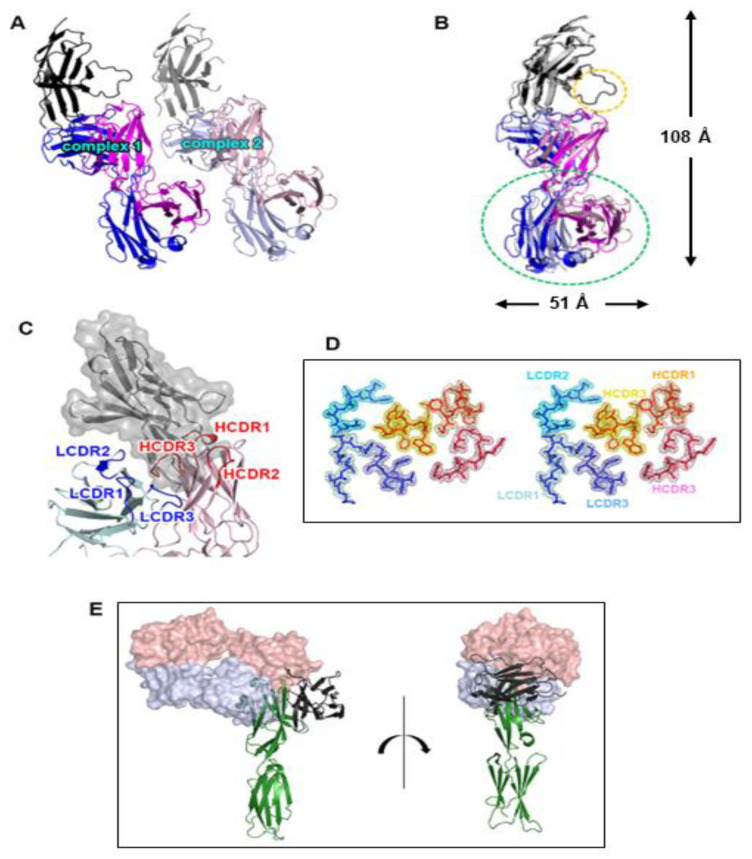
Overall structure of the PD-1/cemiplimab complex. (**A**) Two copies of the complex in an asymmetric unit with a pseudo-translational symmetry. (**B**) Superposition of the two copies for comparison. The displacement of the constant region of the cemiplimab Fab is indicated by a green dotted ellipse. The difference in the C’D loop between the two copies of the complex is indicated by a yellow dotted ellipse. The dimensions of the complex structure are presented. In (**A**) and (**B**), PD-1, heavy, and light chains of cemiplimab are colored black, blue, and purple, respectively. (**C**) The CDR loops of cemiplimab in the complex structure. The CDRs within the light and heavy chains are colored blue and red, respectively. PD-1 is colored gray with a partially transparent surface model. (**D**) Stereoscopic view of 2fofc electron density map on the CDR loops calculated at 1.98 Å resolution and 1.3 σ contour level. (**E**) Superposition of the PD-1 protein within the cemiplimab/PD-1 and PD-L1/PD-1 complexes. Cemiplimab binding blocks the PD-1/PD-L1 interaction through steric occlusion. The cemiplimab Fab is represented as a partially transparent surface model. The heavy and light chains of cemiplimab are colored red and blue, respectively. PD-1 and PD-L1 are colored green and black, respectively.

The ectodomain of PD-1 was expressed as inclusion bodies in *E. coli*, and its soluble form was produced using a refolding procedure. A cemiplimab Fab fragment was expressed in the periplasm of *E. coli*. The 1:1 complex of PD-1 and cemiplimab Fab was present in the solution as a monomer, according to size exclusion chromatography (Supplementary Figure S1). The crystal structure of PD-1 in complex with the Fab fragment of cemiplimab was determined with a resolution of 1.98 Å and *R/R*_free_ of 0.187/0.235. The crystal’s space group is *P1*, and an asymmetric unit contains two copies of the complex with a pseudo-translational symmetric relationship (Figure 1A). Superposition of the structures of PD-1 from the two copies in an asymmetric unit showed that the PD-1 protein and all the six complementarity determining regions (CDRs) of cemiplimab displayed minimal structural differences from one another, with clear electron density on almost all of the complex’s residues (Figure 1B,D). One copy of the complex had a somewhat disordered PD-1 C’D loop, implying that its contribution to cemiplimab binding was likely to be minor (Figure 1B). Due to the inherent flexibility of the elbow region within an antibody, the constant region of the bound Fab was slightly skewed (Figure 1B). Cemiplimab’s PD-1 binding was mediated by five complementarity determining regions (CDRs), leaving LCDR2 uninvolved (Figure 1C). When the complex structure of PD-1 and cemiplimab was overlaid onto the complex structure of PD-1 and PD-L1 with the shared fixed PD-1 (Figure 1E), the underlying mechanism by which cemiplimab blocks the PD-1 from the binding of PD-L1 became clear. The variable region of cemiplimab’s light chain sterically collided with PD-L1 prior to establishing the PD-1/PD-L1 complex when cemiplimab binds to PD-1.

### 3.2. Specific Cemiplimab–PD-1 Interactions

**Figure 2:**
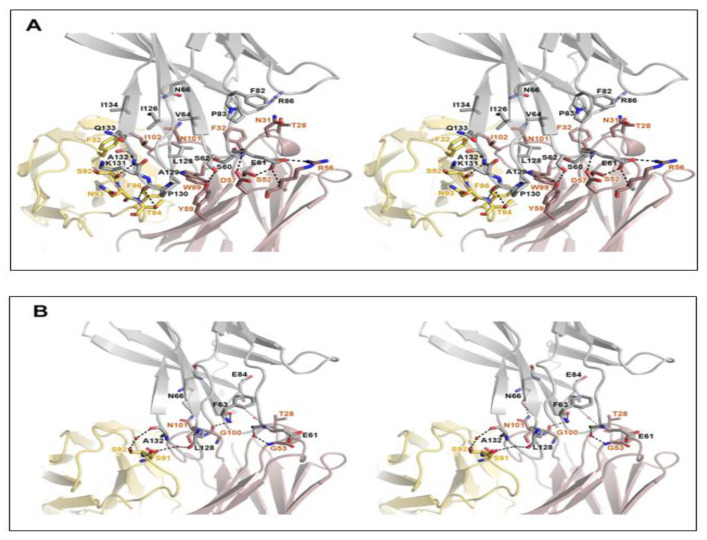
Detailed interactions within the PD-1/cemiplimab interface. (**A**) Stereoscopic view of the interactions, including hydrogen bonds, ionic interactions, and van der Waals contacts between PD-1 and cemiplimab. (**B**) Stereoscopic view of the water-mediated hydrogen bonds. PD-1, heavy, and light chains of cemiplimab are colored gray, pale red, and pale yellow, respectively. Hydrogen bonds and ionic interactions are depicted as dotted lines.

The high-resolution structure of the PD-1/cemiplimab complex made it possible to identify this antibody’s precise epitope and paratope. Although the light chain of cemiplimab contributes mostly to the steric blockage of PD-L1 binding (Figure 1E), a buried solvent-accessible area of 1727 Å^2^ within the binding interface is primarily caused by interactions with the heavy chain (71%). The cemiplimab binding involves 16 residues of PD-1 for the interaction via hydrogen bonds, a salt bridge, and van der Waals interactions (Figure 2). The cemiplimab residues _heavy_S52, _heavy_R56, _heavy_D57, _heavy_Y59, _light_S92, and _light_T94 make hydrogen bonds with the residues of PD-1, particularly _PD-1_E61 and _PD-1_S62 of the BC loop and _PD-1_P130 and _PD-1_A132 of the FG loop (Figure 2A). Furthermore, the side chain of the PD-1 residue _PD-1_E61 also creates a salt bridge with the side chain of _heavy_R56 in cemiplimab heavy chain, implying its crucial contribution to the binding energy of the complex (Figure 2A). The side chains of the cemiplimab residues, including _heavy_T28, _heavy_N31, _heavy_Y32, _heavy_Y59, _heavy_W99, _heavy_N101, _heavy_I102, _light_F32, _light_N93, _light_T94, and _light_F96, make extensive van der Waals contacts with the side chains of the PD-1 residues, including _PD-1_S60 and _PD-1_S62 within the BC loop, _PD-1_P83 and _PD-1_R86 within the C’D loop, _PD-1_L128, _PD-1_A129, _PD-1_P130, _PD-1_K131 and _PD-1_A132 within the FG loop, and _PD-1_V64, _PD-1_N66, _PD-1_F82, _PD-1_I126, _PD-1_Q133, and _PD-1_I134 within the front β-sheet consisting of the CC’FG strands. Thanks to the high resolution of the complex structure, the apparent electron density could visualize the water molecules mediating the hydrogen bonds between the residues of PD-1 and cemiplimab (Figure 2B). The residues of PD-1, including _PD-1_E61, _PD-1_F63, _PD-1_N66, _PD-1_E84, _PD-1_L128, and _PD-1_A132, establish hydrogen bonds via water molecules with _heavy_G53, _heavy_G100, _heavy_N101, _heavy_T28, _light_S91, and _light_S92 of cemiplimab, respectively. A surface plasmon resonance (SPR) study revealed that cemiplimab’s binding affinity (K_D_) to PD-1 is 0.60 nM, whereas the PD-L1 binds to PD-1 with a binding affinity of 8.2 μM [19,31,32]. Taken together, all of the interactions mentioned above would support cemiplimab’s strong affinity to outcompete PD-L1 binding to PD-1.

### 3.3. Remarkably Similar Binding of Cemiplimab and Dostarlimab

**Figure 3:**
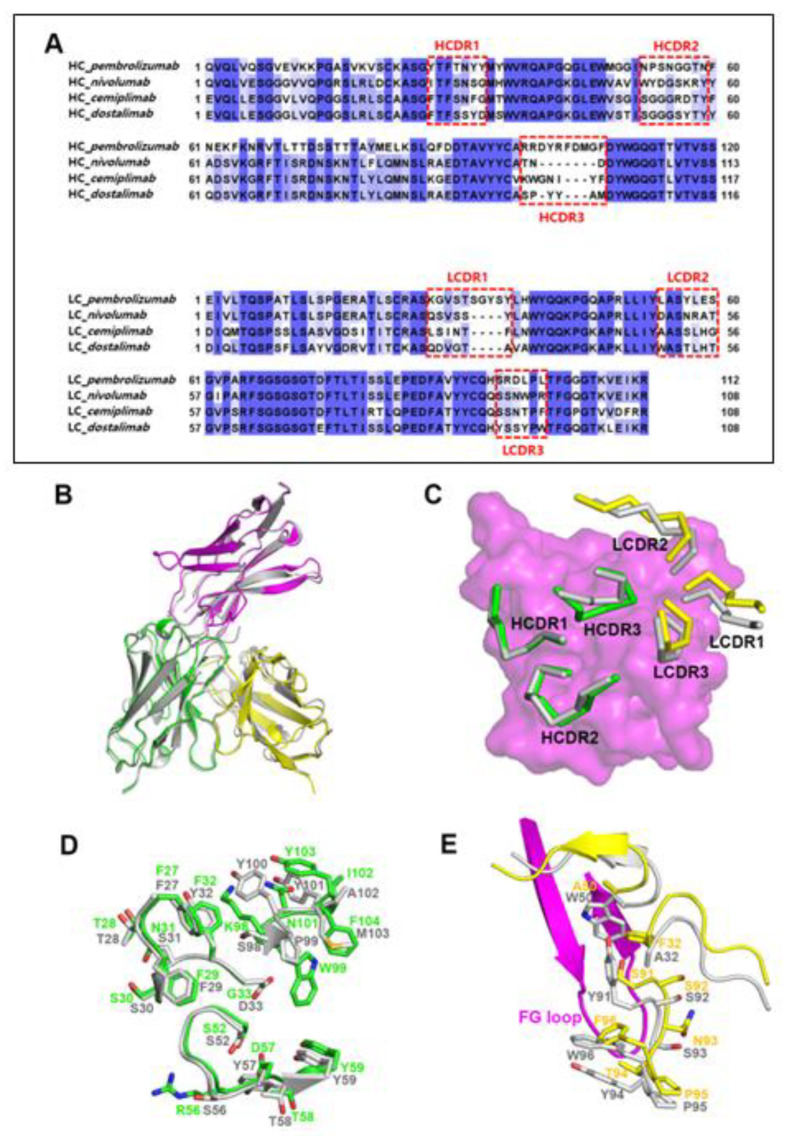
Similar binding modes of cemiplimab and dostarlimab. (**A**) Sequence alignments of the variable regions of the FDA-approved anti-PD-1 antibodies. The CDRs are indicated by dotted boxes. HC and LC mean heavy chain and light chain, respectively. (**B**) Superposition of the PD-1 protein within the cemiplimab/PD-1 and dostarlimab/PD-1 complexes for comparison. The dostarlimab/PD-1 complex is colored gray, and the PD-1, heavy, and light chains in the PD-1/cemiplimab complex are colored purple, green, and yellow, respectively. (**C**) Conformational comparison of the CDRs within the cemiplimab/PD-1 and dostarlimab/PD-1 complexes. The dostarlimab CDRs are colored gray. HCDRs and LCDRs of cemiplimab are colored green and yellow. PD-1 is represented as a purple surface model. (**D**) Detailed structural comparison of the residues within the HCDRs of cemiplimab (green) and dostarlimab (gray). (**E**) Detailed structural comparison of the residues within the LCDRs of cemiplimab (yellow) and dostarlimab (gray). The FG loop of PD-1 in the complex is colored purple.

When the complex structures of PD-1/cemiplimab and PD-1/dostarlimab were superimposed (PDB code 7WSL) [24], it was discovered that their binding modes and epitopes are very similar (Figure 3B). Even though the detailed interactions are somewhat different from each other, the loop conformations of the HCDR1 and HCDR2 remain conserved, most likely as a result of the amino acid sequence similarities within the regions of the two antibodies (Figure 3A). The side chains of _heavy_Y32 and _heavy_D33 within the dostarlimab HCDR1 form hydrogen bonds with PD-1. In contrast, cemiplimab’s corresponding residues, _heavy_F32 and _heavy_G33, are unable to make any hydrogen bonds (Figure 2A and Figure 3D). With the _PD-1_E61 of PD-1, the side chain of _heavy_R56 in the cemiplimab HCDR2 creates a salt bridge, whereas the corresponding residue _heavy_S56 of dostarlimab forms a hydrogen bond. The side chain of _heavy_D57 in cemiplimab HCDR2 forms a hydrogen bond with the backbone nitrogen atom of _PD-1_E61 in PD-1, unlike the analogous residue _heavy_Y57 in dostarlimab. In particular, the HCDR3 was entirely different from one another, with cemiplimab’s HCDR3 being one residue longer than dostarlimab’s (Figure 3A,D). While the residue _light_W50 within the dostarlimab LCDR2 aids in the hydrophobic contact with PD-1’s FG loop area, cemiplimab’s equivalent residue, _light_A50, is significantly smaller than tryptophan and makes little contribution to the interaction (Figure 3E). Instead, the residue _light_F32 within the cemiplimab LCDR1 is important in the interaction with the FG loop, with the dostarlimab residue _light_A32 making a negligible contribution (Figure 2A and Figure 3E). The LCDR3 of both antibodies also participate, albeit in distinct ways, in the interaction with the FG loop. In cemiplimab, hydrogen bonds play a significant role in mediating the interaction between the LCDR3 and the FG loop (Figure 3E). In comparison, the dostarlimab LCDR3 interacts with the FG loop through hydrophobic contacts with the residues _light_Y91 and _light_Y94 [24].

### 3.4. Comprehensive Comparison of the PD-1 Binding by the Antibodies

**Figure 4:**
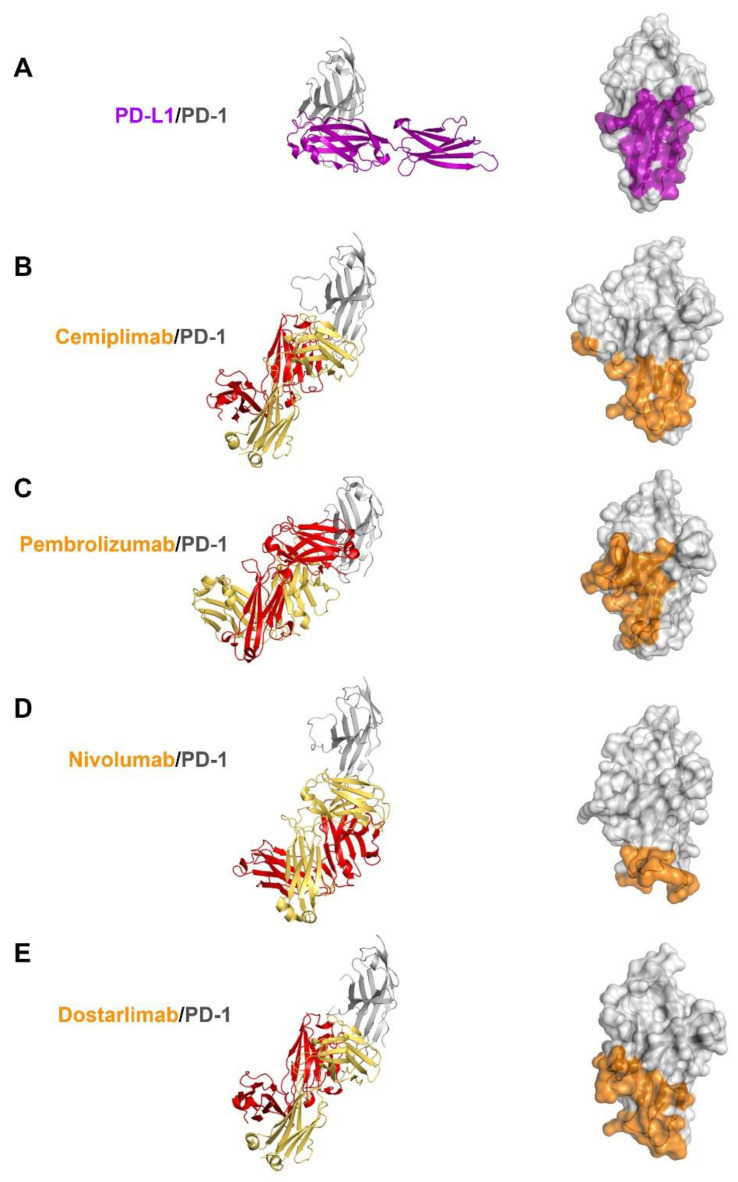
Comparison of PD-1 binding by PD-L1 and antibodies. (**A**) Structure of the extracellular domain of PD-1 (gray) in complex with the extracellular PD-L1 (purple) and the PD-L1 binding site (purple) on the surface of PD-1. (**B**) Structure of the extracellular domain of PD-1 in complex with the cemiplimab Fab and its epitope region on the surface of PD-1. (**C**) Structure of the extracellular domain of PD-1 in complex with the pembrolizumab Fab and its epitope region on the surface of PD-1. (**D**) Structure of the extracellular domain of PD-1 in complex with the nivolumab Fab and its epitope region on the surface of PD-1. (**E**) Structure of the extracellular domain of PD-1 in complex with the dostarlimab Fab and its epitope region on the surface of PD-1. In (**A**–**E**), PD-1 is displayed in the same orientation, and the heavy and light chains of the antibodies and their epitope regions are colored red, yellow, and orange, respectively.

**Figure 5:**
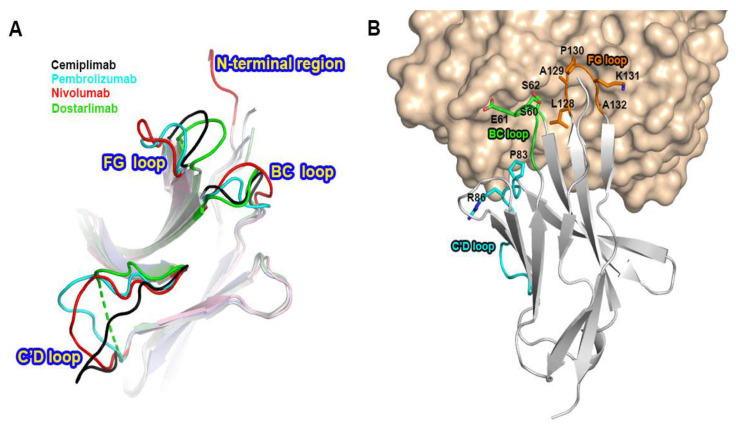
Conformational changes of the PD-1 loops by antibody-binding. (**A**) Superposition of PD-1 extracted from its complex structures with the FDA-approved antibodies shows the diverse conformations of the loops within PD-1 by antibody-binding. (**B**) The involvement of the PD-1 loops, including the BC, C’D, and FG loops, in the interaction with cemiplimab. PD-1 is presented as a gray ribbon model (), and cemiplimab as a yellow surface model. The BC, C’D, and FG loops are colored green, cyan, and orange, respectively. The residues within the PD-1 loops, which are involved in cemiplimab-binding, are represented as stick models.

Previous studies have reported the complex structures of PD-1 with the FDA-approved antibodies, nivolumab, pembrolizumab, and dostarlimab, describing the structural basis of PD-1 blockades by antibody drugs [20,21,22,23,24]. The molecular mechanism underlying the clinical efficacy of the PD-1 antibodies can be better understood by comparing the binding interactions of PD-1 with PD-L1 and the antibodies in extensive detail. The antibodies and PD-L1 bind to the immunoglobulin-like extracellular domain of PD-1 from various directions and on distinct binding sites when the PD-1 structures extracted from the complex structures with PD-L1, cemiplimab, pembrolizumab nivolumab, and dostarlimab were superimposed (Figure 4). The binding of PD-1 to PD-L1 is mediated by the interaction involving the residues within the front β-sheet of the ectodomain of PD-1, while the antibody drugs predominantly interact with the loop regions of PD-1 protein [23,29]. Although the area of the binding interface between PD-1 and PD-L1 is comparable to the interfaces of antibody-binding, the antibody drugs bind to PD-1 with considerably increased binding affinity than PD-L1, implying the loop regions would be hotspots on PD-1 for effective interaction between PD-1 and the antibody drugs. Although there is no head-to-head comparison of the antibodies’ effect in vitro, the binding affinities (Kd) of the antibodies to PD-1 vary; 27 pM for pembrolizumab, 4.06 nM for nivolumab, 0.6 nM for cemiplimab, and 0.3 nM for dostarlimab [17,19,21,22].The loops of the PD-1 ectodomain adopt distinct structures when they engage to various binding partners, such as PD-L1 and the antibody drugs, implying that they are intrinsically flexible and their optimal conformations are induced to increase binding affinity (Figure 5). The binding of nivolumab is largely dependent on its ability to interact with the N-terminal region of PD-1, whereas pembrolizumab and dostarlimab bind primarily to the C’D loop of distinct conformation [20,21,22,23,24]. The nivolumab-bound form of PD-1 was the only crystal structure in which the N-terminal extension of PD-1 was visible, suggesting its inherent flexibility. The C’D loop encroaches upon a groove created by the paratope of pembrolizumab, and the majority of the loop’s residues participates in the interaction with the drug. The C’D loop would also be the primary contributor to the dostarlimab-binding of PD-1 because the residue R86 in the C’D loop of PD-1 occupies a concavity within the paratope of dostarlimab through hydrogen bonds, ionic interactions, and pi–cation interactions. Additionally, the BC and FG loops of PD-1 also undergo conformational changes upon binding to these antibodies, providing extra interactions with them [20,24]. Additionally, these loops’ specific conformations are incompatible with PD-L1 binding because they participate in the PD-L1 interaction in distinct conformations. Even though cemiplimab and dostarlimab similarly bind to PD-1, the differences in the specific interaction mentioned above caused the loops’ conformation to vary for the best interaction with the two antibodies. The binding of cemiplimab to PD-1 also involves the interaction with the flexible BC, C’D, and FG loops of PD-1. Given that the PD-1 residue E61 is thought to play a significant role in the interaction with cemiplimab through a salt bridge and multiple hydrogen bonds, the BC loop of PD-1 would be the dominant hotspot for binding cemiplimab.

## 4. Discussion

Blocking the interaction between PD-1 and PD-L1 by monoclonal antibodies removes the ability of tumors to evade the immune system, revolutionizing cancer immunotherapy. Among the FDA-approved antibodies, the most redundant therapeutic approach is blocking the PD-1/PD-L1 interaction, with four anti-PD-1 antibodies, including pembrolizumab, nivolumab, cemiplimab, and dostarlimab, and three anti-PD-L1 antibodies, including atezolizumab, avelumab, and durvalumab [33]. 

In this study, we describe the high-resolution crystal structure of the PD-1/cemiplimab complex. Overall, the binding of cemiplimab is very distinct from pembrolizumab and nivolumab but strikingly resembles that of dostarlimab. Cemiplimab is the third anti-PD-1 antibody approved by the US FDA for the treatment of metastatic cutaneous squamous cell carcinoma in 2018. In 2021, the FDA approved this antibody as the first immunotherapy for locally advanced basal cell carcinoma. In addition, ceimplimab monotherapy was also approved as a first-line treatment for patients of advanced non-small cell lung cancer with high PD-L1 expression. The fourth PD-1 monoclonal antibody, dostarlimab was approved by the FDA for the treatment of mismatch repair deficient (dMMR) endometrial cancer and dMMR solid tumors in 2021. Our study reveals that the binding modes of cemiplimab and dostarlimab are strikingly similar except for some details. Besides, these two antibodies are both IgG4 kappa antibodies, expecting their negligible effector functions, including ADCC and CDC activity. However, their approved indications are not redundant, so additional approvals of their indications and combination therapies with these antibodies are anticipated. Recently, relatlimab, an anti-LAG-3 antibody, was approved to treat melanoma in combination with nivolumab [34]. This approval is especially notable since it validates a completely novel combination of two immunotherapies that may work in tandem to enhance anti-tumor response by targeting LAG-3 and PD-1, two separate immune checkpoints. Cemiplimab is also under clinical investigation in combination with a LAG-3 blocking antibody fianlimab, demonstrating a greater than 60% response rate in melanoma patients [35]. To date, no structural information for the antibody-mediated blockade of LAG-3 is available. Further structural studies of LAG-3 antibodies can provide more details on the molecular mechanism of the therapeutic synergy of the dual blockade of PD-1 and LAG-3.

Due to their exceptional specificity and affinity to their targets, monoclonal antibodies have become a major therapeutic tool. Different epitopes might result in varying therapeutic efficacies for antibodies, so the epitope inside a target molecule can be a key component of a therapeutic antibody. The authorized antibodies against PD-1 and PD-L1 detect different antigenic epitopes from other antibodies, even though they share a similar blocking mechanism [25]. In this study, cemiplimab has a different binding mode from other antibodies and mainly interacts with the flexible loops of PD-1, including the BC, C’D, and FG loops. These structural investigations into the hotspots on a target protein where antibodies bind may help develop more effective biologics against PD-1 for cancer immunotherapy. The observation and study of off-target effects caused by antibodies on huge cohorts have revealed that they are not as specific as previously believed, raising new concerns about the polyreactivity and polyspecificity of antibodies [36,37]. Accumulating structural information for the recognition of various targets by antibodies could also contribute to enhancing the target specificity of therapeutic antibodies.

## 5. Conclusions

In conclusion, the high-resolution structure of PD-1 in complex with the Fab fragment of cemiplimab sheds light on the molecular mechanism underlying the therapeutic activity of this antibody drug. Additionally, the specific epitope and interactions revealed by this structural study may provide significant information for the improvement of the current biological agents against PD-1 for cancer immune checkpoint therapy.

## Figures and Tables

**Table 1:** Data collection and refinement statistics.

Data Collection	
X-ray source	PLS 5C
Wavelength (Å)	1.0000
Space group	P1
a, b, c (Å)	55.06, 62.96, 81.29
α, β, γ (˚)	105.73, 98.00, 92.44
Resolution (Å)	1.98 (2.05–1.98) ^1^
R_meas_ (%)	7.20 (64.1)
I/σI	10.95 (2.21)
Completeness (%)	94.7 (94.6)
Redundancy	2.8 (2.5)
CC_1/2_	0.998 (0.760)
Refinement	
Resolution (Å)	1.98
No. of reflections	68,386
R_work_/R_free_ (%)	18.7/23.5
No. atoms	
Protein	8210
Water	644
Average B-factor (Å^2^)	38.0
R.m.s. deviation	
Bond lengths (Å)	0.008
Bond angles (˚)	1.039
Ramachandran	
Favored (%)	97.63
Allowed (%)	2.37
Outlier (%)	0.00
PDB code	8GY5

^1^ Values in parentheses are for the outer resolution shell.

## Data Availability

The atomic coordinates and structure factors for the complex structure of PD-1/cemiplimab were deposited in Protein Data Bank (http://www.rcsb.org (deposited: 21 September 2022)) under the accession code 8GY5.

## Supplementary Materials

The following supporting information can be downloaded at: https://www.mdpi.com/article/10.3390/biomedicines10123154/s1, Figure S1: Preparation of the protein of PD-1/cemiplimab complex.
