# Supplementary material for: The High-Resolution Structure Reveals Remarkable Similarity in PD-1 Binding of Cemiplimab and Dostarlimab, the FDA-Approved Antibodies for Cancer Immunotherapy
Source: Biomedicines. 2022 Dec 6;10(12):3154. doi: 10.3390/biomedicines10123154 (PMC9775377; doi:10.3390/biomedicines10123154)
Supplement: Supplementary file 1 [file biomedicines-10-03154-s001.zip › biomedicines-2039502-supplementary.pdf]

SUPPLEMENTARY MATERIALS

The High-resolution Structure Reveals Remarkable Similarity in PD-1 Binding of Cemiplimab and Dostarlimab, the FDA-approved Antibodies for Cancer Immunotherapy

Tae Jun Jeong[†], Hyun Tae Lee[†], Nahyeon Gu, Yu-Jeong Jang, Seung Beom Choi, Ui Beom Park, Sang Hyung Lee and Yong-Seok Heo[*]

Expression and Purification of Proteins

Genes encoding the ectodomains of human PD-1 (aa 26-150) was subcloned into pET-21a and the protein was expressed in *E. coli* BL21(DE3) as inclusion bodies. The cells were grown at 37 ˚C in LB medium supplemented with 50 μg mL^{-1} ampicillin until OD_{600} reached 0.6-1.0, and the protein expression was induced with 1mM IPTG and incubated for 4 h at 37 ˚C. The cells were harvested by centrifugation, re-suspended in lysis buffer (20 mM Tris, pH 8.0, 200 mM NaCl) and lysed by sonication on ice. Inclusion bodies were recovered by centrifugation (25,000 × g for 0.5 h at 4°C) and solubilized in 8 M urea, 20 mM Tris, pH 8.0, 200 mM NaCl by stirring overnight. After removing undissolved residue by centrifugation (25,000 × g for 0.5 h at 4°C), solubilized fraction was applied to HisTrap HP column and washed with five column volumes of wash buffer (8 M urea, 20 mM Tris, pH 8.0, 200 mM NaCl, 50 mM imidazole). The protein was then eluted with elution buffer (8 M urea, 20 mM Tris, pH 8.0, 200 mM NaCl, 400 mM imidazole). The eluted PD-1 protein was refolded by dialysis 3 times against 20 mM Tris, pH 8.0, 200 mM NaCl and purified further by gel filtration chromatography using a HiLoad 16/60 Superdex 200 pg column.

The DNA sequence for the Fab fragment of cemiplimab was synthesized after codon-optimization for expression in *E. coli*. The sequences for heavy chain and light chain were cloned into a modified pBAD vector, containing the STII signal sequence in each chain for periplasmic secretion and a C-terminal 6His-tag in heavy chain. The plasmid pBAD-Fab was transformed into *E. coli* Top10F. The cells were grown at 37 ˚C in LB medium supplemented with 50 μg mL^{-1} ampicillin. At an OD_{600} of 1.0, the protein expression was induced with 0.2% arabinose and cells were grown at 30 °C for 15 h. The cells were harvested by centrifugation, re-suspended in lysis buffer (20 mM Tris, pH 8.0, 200 mM NaCl) and lysed by sonication on ice. After removing cell debris by centrifugation (25,000 × g for 0.5 h at 4°C), the supernatant

containing soluble protein was applied to HisTrap HP column and washed with five column volumes of wash buffer (20 mM Tris, pH 8.0, 300 mM NaCl, 50 mM imidazole). The protein was then eluted with elution buffer (20 mM Tris, pH 8.0, 300 mM NaCl, 400 mM imidazole). The eluted protein was concentrated for gel filtration chromatography using a HiLoad 16/60 Superdex 200 pg column. The column had previously been equilibrated with gel filtration buffer (20 mM Tris, pH 8.0, 300 mM NaCl).

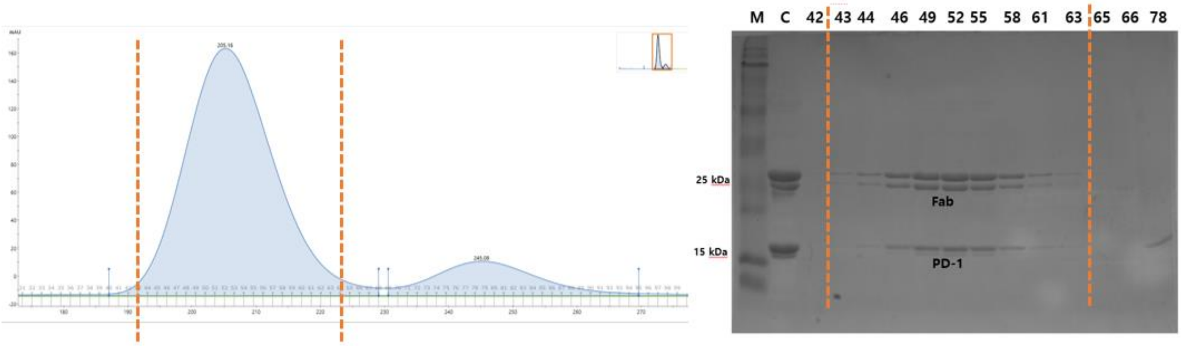

Supplementary Figure 1. Preparation of the protein of PD-1/cemiplimab complex.

Gel-filtration chromatography profile and SDS-PAGE analysis of the protein of PD-1/cemiplimab complex. The fractions between the two vertical bars are collected for crystallization
